# Optoacoustically induced auditory brainstem responses in the mouse model enhanced through an absorbing film

**DOI:** 10.1117/1.JBO.26.9.098001

**Published:** 2021-09-03

**Authors:** Katharina Sorg, Larissa Heimann, Gabriela Moreira Lana, Achim Langenbucher, Bernhard Schick, Eduard Arzt, Gentiana Ioana Wenzel

**Affiliations:** a Saarland University Medical Center, Department of Otorhinolaryngology, Homburg, Germany; b INM Leibniz Institute for New Materials, Saarbrücken, Germany; c Saarland University, Department of Materials Science and Engineering, Saarbrücken, Germany; d Saarland University, Department of Experimental Ophthalmology, Homburg, Germany

**Keywords:** optoacoustic, laser, auditory brainstem responses, silicone elastomers, optoacoustically induced auditory brainstem responses

## Abstract

**Significance:** Optoacoustic stimulation offers an alternative stimulation strategy for the hearing organ. To serve as the base for a novel auditory prosthesis, the optoacoustic stimulation must be biocompatible and energy-saving.

**Aim:** Enhancing the efficiency of optoacoustic stimulation while reducing the energy input in a suited animal model.

**Approach:** Optoacoustically induced auditory brainstem responses (oABRs) were recorded after the pulsed laser irradiation of the tympanic membrane (TM) in mice. The results were compared with the ABRs induced through acoustic click stimulation. In addition, self-adhesive absorbing films were applied on the TM before the optoacoustic stimulation to investigate their effect on the resulting ABRs.

**Results:** Using an absorbing film on the TM during optical stimulation led to considerably enhanced oABR wave I amplitude values compared with the stimulation of the bare TM. When using our stimulation strategy, we induced oABR waves in the 50% to 60% range of the acoustical stimulation reached with 80-dB SPL click stimuli.

**Conclusions:** The mouse model can be used for certain developmental work for an optoacoustic auditory prosthesis. Using absorbing films on the TM during optical stimulation considerably enhances oABR wave I amplitude. Optimization of the stimulation strategy could further enhance the efficiency within biocompatibility margins.

## 1 Introduction

Optoacoustics is applied in the fields of, e.g., imaging, spectroscopy, and quantification of molecules. The result of the absorption of pulsed light in an absorber medium inducing a thermal expansion and contraction of the substrate and, therefore creating a sound source, represents the optoacoustic effect. This effect can be used to stimulate the hearing organ by inducing vibrations of the irradiated structures. Since optoacoustic stimulation could be applied on every stimulation target in the peripheral hearing system, which could vibrate, e.g., the eardrum or the otic capsule, it offers the potential for use in the development of a new generation of auditory prostheses.

The optoacoustic-induced vibrations are transmitted through the auditory pathway and activate the central auditory system. This activation can be analyzed by recording auditory brainstem responses (ABRs) to the optoacoustic stimuli. ABRs are electroencephalographic signals recorded through peripheral electrodes detecting voltages generated by neural activity throughout the brain, including the auditory brainstem and the eighth cranial nerve.^1^ ABR waves can either be induced by acoustical (aABR), electrical (eABR), or optoacoustic (oABR) stimulation and are a well-established method to analyze the hearing function for different basic and advanced research purposes.^2^^–^^6^ In 2009, Wenzel et al.^7^ first demonstrated that optoacoustic stimulation in the peripheral hearing system induces ABR waves in guinea pigs, resembling the form of acoustically induced waves. In a further study, we were able to demonstrate a novel stimulation strategy to induce frequency-specific optoacoustic vibrations in the tympanic membrane (TM), demonstrated by evoked activities in the inferior colliculus in guinea pigs.^8^ Recently, we demonstrated that the effectiveness of optoacoustically induced vibrations of the guinea pig TM depends on the laser wavelength, most probably through dissimilar absorption characteristics of the TM tissues for different wavelengths.^9^ However, one main work package that needs to be performed, before considering the design of a hearing device, is the optimization of the stimulation method to achieve higher activation intensities within biocompatibility margins. We, therefore, sought to assess if the induced vibrations can be amplified by the application of highly absorbing material on the target-irradiated structure in an animal model.

So far, biocompatibility studies have been performed in mice^10^ due to better availability of suited immunostaining antibodies on the market for this animal model compared with bigger mammals, e.g., gerbils or guinea pigs. Electrophysiological studies for optoacoustic stimulation have been, however, performed in guinea pigs.^7^^,^^8^ To directly use the safety margins defined in our previous biocompatibility report,^10^ we decided for the herein presented set of experiments to use mice and needed therefore to establish as well our stimulation strategy for the murine TM as well.

For the highly absorbing material, we chose at this stage silicone elastomers that are promising biomaterials with a broad range of applications.^11^^–^^14^ Among the different silicones, a subclass of poly-(dimethylsiloxane) (PDMS) elastomers, the pressure-sensitive adhesives present interesting properties for applications such as skin adhesives.^12^^–^^15^ They adhere steadily after a short contact time and with low applied pressure on biological surfaces. As they have a low elastic modulus, they can adapt to the surface and reach high adhesion through Van der Waals interface interactions, dismissing the use of adhesive glues or any chemical fixation.^16^ Recently, we were able to introduce soft skin adhesive (SSA) as a promising material for wound scaffolding with high cellular biocompatibility, good adhesion on rough surfaces, such as human skin, and gentle peel-off characteristics, without damaging the tissue. The gentle attachment and detachment are essential for the application onto sensitive tissues, e.g., the TM.^17^^,^^18^ PDMS grafts were successfully used in the treatment of human TM perforations^19^ and demonstrated to have similar basic acoustic properties in the higher frequency range that replicate the human TM motion.^20^

Therefore, to further improve the optoacoustic stimulation, we analyzed herein these silicone elastomers, further named films, as the absorbing material. To analyze the electrophysiological effects of this stimulation method, we assessed, to our knowledge for the first time in literature, the optoacoustic-induced auditory brainstem responses (oABR) in a mouse model.

## 2 Materials and Methods

### 2.1 Animal Model

We used 4- to 12-weeks old female CBA/J mice (Janvier Labs, France) weighing 18 to 23 g in our experiments (14 animals in total in this study). The studies were performed according to the guidelines of the Animal Care and Use Committee of Saarland by qualified persons, approved by the Animal Welfare Agency under the State Office for Consumer Protection of Saarland. All animals were initially anesthetized intraperitoneally with 100 mg/kg ketamine (Ketaset, Zoetis, Berlin; Germany) 10 mg/kg xylazine (Rompun, Bayer, Leverkusen; Germany). The anesthesia was maintained by injection of ¼ to ½ of the initial dose intraperitoneally every 30 min. To keep the body temperature constant at 37°C, the animals were positioned on an electrical heating pad throughout the experiment and were supplied with additional oxygen. To expose the TM of mice for film application and subsequent irradiation, the outer ear canal had to be prepared. Therefore, the hair around the outer ear canal was trimmed and a vertical incision beginning at the incisura intertragica expanding along the cartilaginous outer ear canal was made. The TM was exposed by fixing the edges of the incision with sutures.

### 2.2 Film Application

**Fig. 1:**
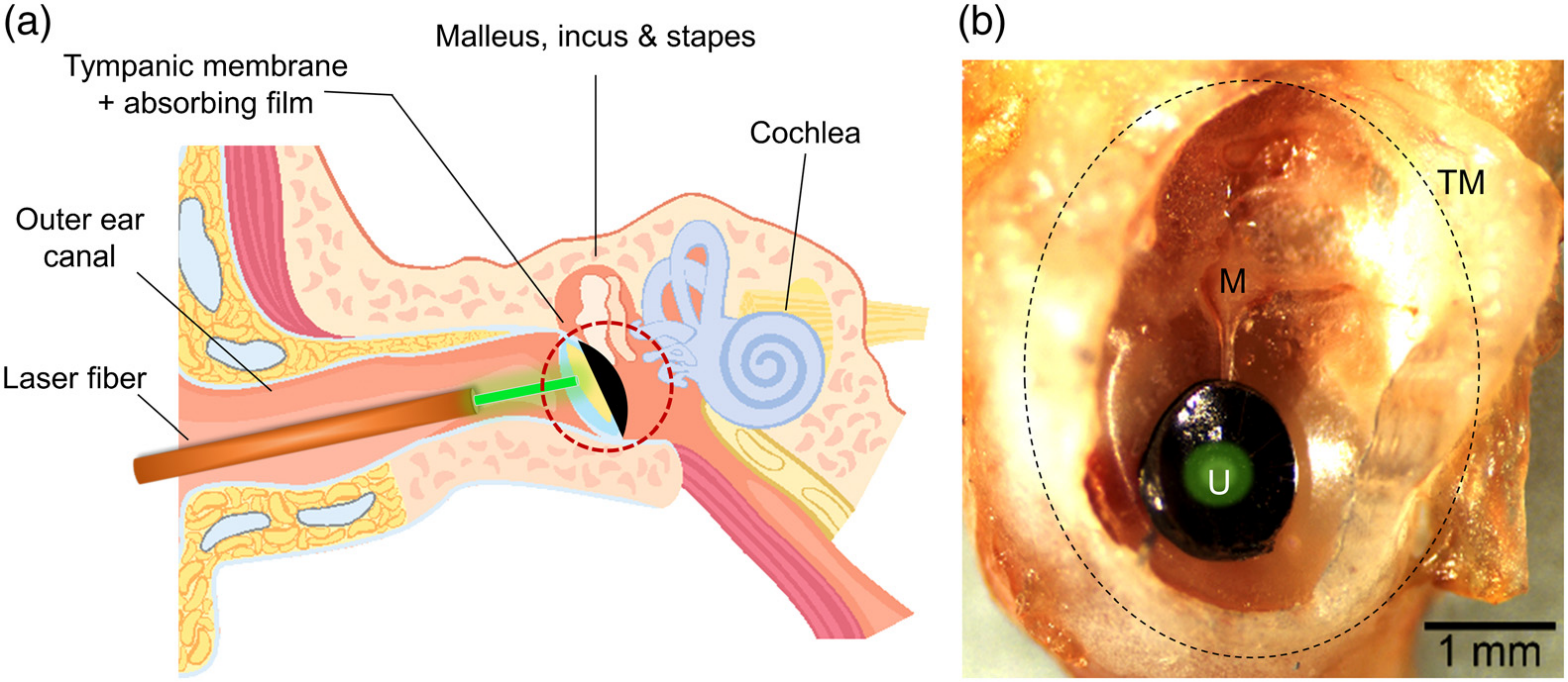
Schematic drawing for the position of the laser fiber inside the outer ear canal (a) centrally over the absorbing patch attached to the TM. (b) Detailed information of the red circled area in (a) showing the absorbing film applied on an explanted murine TM (black dotted circle) with a graphical illustration of the green laser spot in the center of the absorbing film (round black structure) covering the umbo (U) the deepest point of the malleus (M). The scale bar in (b) represents 1 mm. The image in (a) was purchased and edited from iStock.com/iLexx.

**Fig. 2:**
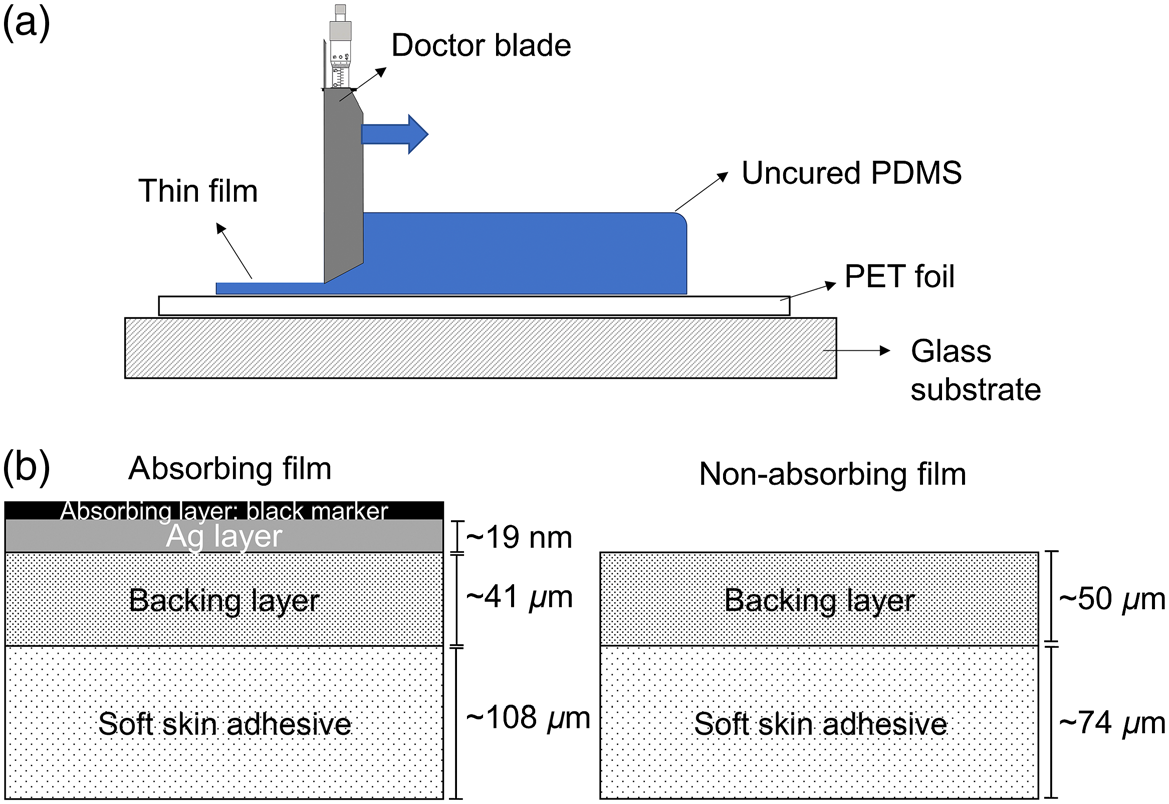
(a) The films were produced using the doctor blade technique as double layer design of SSA and backing layer. (b) Two types of patches were produced: (i) absorbing films with an Ag layer and an absorbing layer of black marker on top of the backing layer, having a total thickness of ∼150  μm and (ii) pristine patches without any absorbing layer, with a total thickness of ∼125  μm.

The films (Fig. 2) were punched with a suction tube manually to ∼1  mm diameter under microscope control and applied carefully with forceps, with the adhesive side in contact to the TM centrally over the umbo [Fig. 1(b)]. We covered the TM with films of different constitutions: (i) transparent (nonabsorbing) films, (ii) absorbing films, and compared the results to (iii) mice that were irradiated without the use of any film (control). The absorbing films were covered with an additional layer of sputtered silver that was then stained with black color (permanent marker, edding International GmbH, Ahrensburg, Germany) to ensure increased laser light absorption. The silver coating is meant to ensure the total reflection of light that could otherwise pass through the absorbing layer. The films were placed on both TM’s of the animal: one side for the irradiation and the other side served as a control.

### 2.3 Laser Irradiation

The laser irradiation was always performed on the left ear of the mice. We used a 532-nm pulsed neodymium-doped yttrium orthovanadate (${\mathrm{Nd}:\mathrm{YVO}}_{4}$) laser (Xiton Photonics GmbH, Kaiserslautern, Germany) as the light source. The pulsed laser light was applied through a glass fiber (365  μm diameter, FG365LEC-CUSTOM, Thorlabs GmbH, Munich, Germany) directed with a micromanipulator (Narishige, Tokyo, Japan) under microscope control vertically to the surface and in the center of the absorbing film or TM [Fig. 1(a)]. The distance to the irradiated structure was ∼300 to 500  μm. Therefore, the calculated irradiation spot diameter was ∼590  μm, whereas the laser spot was either positioned centrally over the absorbing or nonabsorbing film or the native TM at the umbo [Fig. 1(b)]. The laser irradiation parameters were chosen to replicate our self-designed stimulation strategy creating a sinusoid signal at the targeted frequency, the laser-modulation rate (LMR). We irradiated the selected area for 5 ms and paused for 95 ms with average laser powers of 2, 3, 5, 8, 12, 20, 31, 50, and 79 mW with the laser repetition rate (LPR) of 50 kHz and LMRs of 1, 8, and 10 kHz. Afterward, the laser power was calibrated using a power meter (Uno, Gentec Electro-Optics, Inc., Québec, Canada).

### 2.4 Electrophysiology: aABR and oABR

Before and after the patch application, as well as after the laser irradiation, click auditory brainstem response (click ABR) recordings were performed to assess the hearing function of the mice and serve as controls for the effectiveness of optical stimulation. The recording of ABRs was performed in the same way as previously reported.^3^^,^^21^^–^^23^ We recorded ABRs using subcutaneous needles: one on the mastoid, one at the vertex (reference), and one at the base of the tail (ground). The click stimuli were generated with a digital signal processing system (Agilent 33500 B Series True form Waveform Generator, Keysight Technologies GmbH, Germany) and were delivered through a free field speaker (custom made from a DT-911, Beyerdynamic GmbH & Co. KG, Germany^3^) placed in a 5-cm distance in front of the left ear (the irradiated ear). The recorded signals were then amplified through the biosignal amplifier (g.USBamp, g.tec medical engineering GmbH, Austria), digitized at 19.2 kHz, and filtered to obtain the frequencies from 300 to 2500 Hz. The stimulus intensities ranged from 0 to 80 dB SPL, increased in 10 dB steps. The stimulus repetition rate was 20 Hz, and 500 trials were averaged. The speaker output was calibrated periodically. The hearing thresholds were determined visually during the recording as well as offline and were defined by the lowest intensity where the Jewett’s wave complex was identifiable. The Jewett complex was first described by Jewett and Williston in 1971.^24^ In mice, it typically consists of five vertical positive waves between 1 and 6 ms.^25^^–^^27^ During the laser irradiation, we recorded ABR signals that were generated by laser-induced stimulation (oABR) from 2 to 79 mW for each LMR. We analyzed wave I amplitude [Fig. 4(a)] after acoustic (aABR) and after laser stimulation (oABR). The amplitude was determined as the absolute value between the first negative ($I_{n}$) and first positive ($I_{p}$) values of the first wave [Fig. 4(a)]. We normalized the resulting oABR amplitudes at the respective laser stimulation levels to the maximum reached aABR amplitude at 80 dB SPL of each animal and averaged the resulting data between the animals in the three different groups. To analyze the o- and aABR signals at different energy levels of the stimulus, the measured values were fitted with a sigmoidal function. Information about data fitting is included in the Supplementary Material.

### 2.5 Production and Characterization of the Silicon Films

The silicone films used in this study consisted of multilayer samples, prepared as follows [see Fig. 2(a)]. First, a polydimethylsiloxane (PDMS, Elastomer kit Sylgard 184, Dow Silicones, Midland, Michigan) film was manufactured on a polyethylenterephthalat (PET) foil by the doctor blade technique using an automatic film applicator (MSK-AFA-IV, MTI Corporation, Richmond, California) set with a gap of 100  μm. The film was then cured at 95°C for 1 h. Subsequently, on the cured Sylgard 184 film, a second layer was prepared, of SSA MG7-1010 (Dow Silicones, Midland, Michigan), also using a doctor blade, set for 200 or 300  μm in total (cured Sylgard + SSA). The double-layer sample was removed from the PET foil and placed on a fluorosilicone release foil (3M^TM^ Scotchpak^TM^ 9709 Release Liner) with the SSA surface (adhesive side) on the foil for further use. Two different designs of samples were prepared [Fig. 2(b)]: (i) pristine patches without any absorbing layer and (ii) patches covered with a thin Ag layer on the PDMS side and a layer of black ink above that. For the second design, a thin Ag layer was deposited on the Sylgard surface (JEOL-1300 Auto fine coater) under vacuum using 30 mA for 180 s. Covering the Ag layer, a black film was prepared using, as described before, a black marker (permanent marker, edding International GmbH, Ahrensburg, Germany) [Fig. 2(b)]. For further characterization, the thickness of the polymer films was measured using an optical microscope (Eclipse LV100ND, Nikon, Tokyo, Japan), and the transmission spectrum of the samples was recorded by UV–Vis spectrometry (Cary 5000 UV-Vis-NIR, Agilent, Santa Clara, California) in the range from 200 to 1300 nm. To determine the thickness of the Ag layer, ellipsometric spectroscopy (J.A. Woollam Co., Lincoln, Nebraska) was used, varying the incidence angle from 65 deg to 75 deg in 5 deg steps, and averaging 50 measurements. The following data analysis was performed according to the Cauchy Model with the Software WVASE32 from Woollam.

### 2.6 Statistical Analysis

For statistical analysis, OriginPro 2020 software was used (OriginLab Corp., Northhampton, Massachusetts). The Shapiro-Wilk-test was applied to verify the normal distribution of the data followed by a Levene’s test for variance homogeneity. For the analysis of aABR amplitude before and after patch application, we performed paired t-tests at each acoustical stimulation level.

## 3 Results

### 3.1 Production and Characterization of the Silicon Patches

**Fig. 3:**
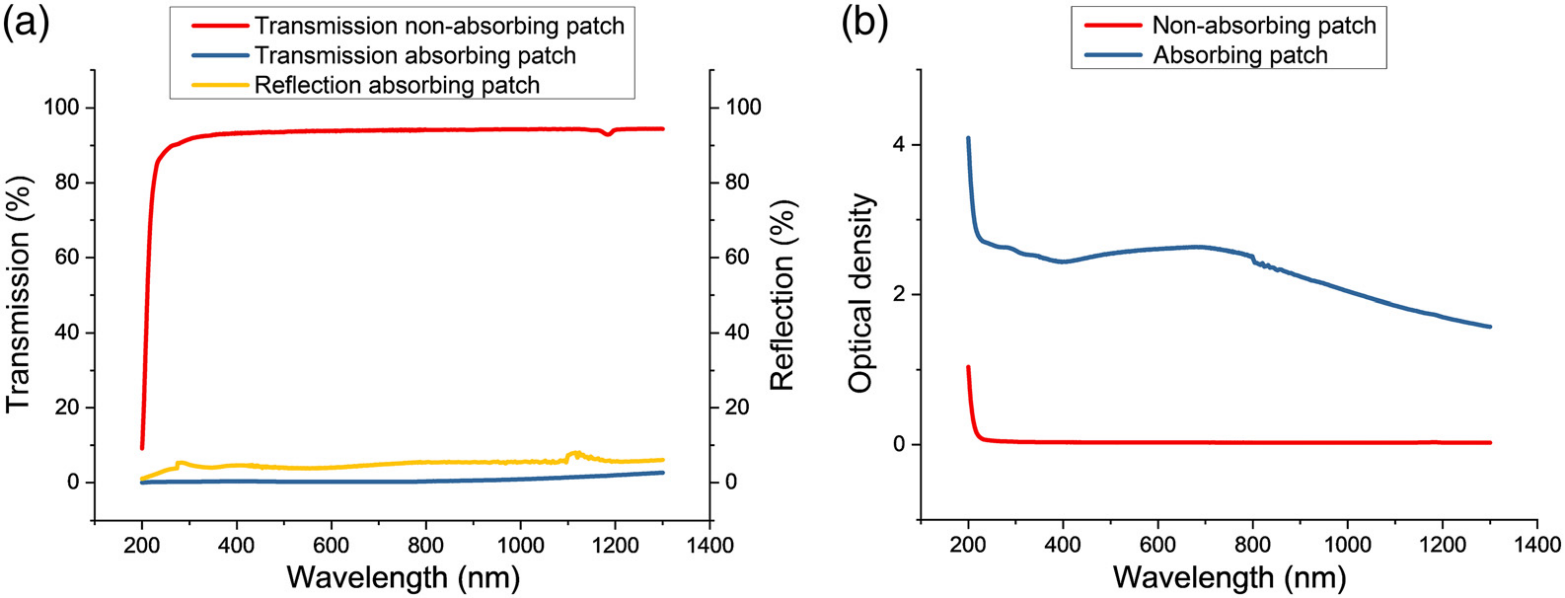
(a) Transmission, reflection, and (b) optical density spectra of absorbing and nonabsorbing films between 200 and 1300 nm.

First, the absorbing film, containing the Ag and the black absorbing layers, as well as the nonabsorbing sample, the pristine PDMS-SSA double layer, were measured regarding its dimensions, light transmission, and absorbance. For the nonabsorbing film, the SSA portion was 74.16±2.06  μm thick, and the PDMS backing layer was 49.82±1.35  μm. The absorbing film consisted of an SSA film of 108.26±16.42  μm, a PDMS backing layer of 41.53±1.79  μm, and the Ag thin film of 19.9 nm thickness, covered by the black absorbing surface. As described in Sec. 2.1.1, the films were punched with a suction tube in circular patches and positioned on the TM [Fig. 1(b)]. The transmission and reflection spectra obtained from UV–Vis spectrometry are shown in Fig. 3(a), in which the transmission of the nonabsorbing samples demonstrated a plateau of ∼94% for wavelength values above 300 nm, being 93.72% at 532 nm. The absorbing structure, on the other hand, demonstrated a constant behavior of low transmission, being about 0.24% at 532 nm. This behavior was attributed to the combination of the black and silver layers. Measurements of the silver layer before staining the film black demonstrated partially blocking the transmission of the incident irradiation. The silver layer combined with the absorbing layer could achieve even lower transmission (under 0.5%), as shown in Fig. 3(a), minimizing the transference to the TM. The reflection of the absorbing patch is also presented and lies at ∼5% or less over the measured wavelength range, being 3.843% at 532 nm. The optical density of these samples at the different wavelengths [Fig. 3(b)] was calculated as Abs=log(1/T) and was at 532 nm 2.574 for the stained absorbing structure and 0.028 for the control film. These values take into account both absorption as well as the reflection of the samples. Considering the incident radiation as the sum of absorption, reflection, and transmission, we obtained absorption values of the absorbing film of ∼95%.

### 3.2 oABR

**Fig. 4:**
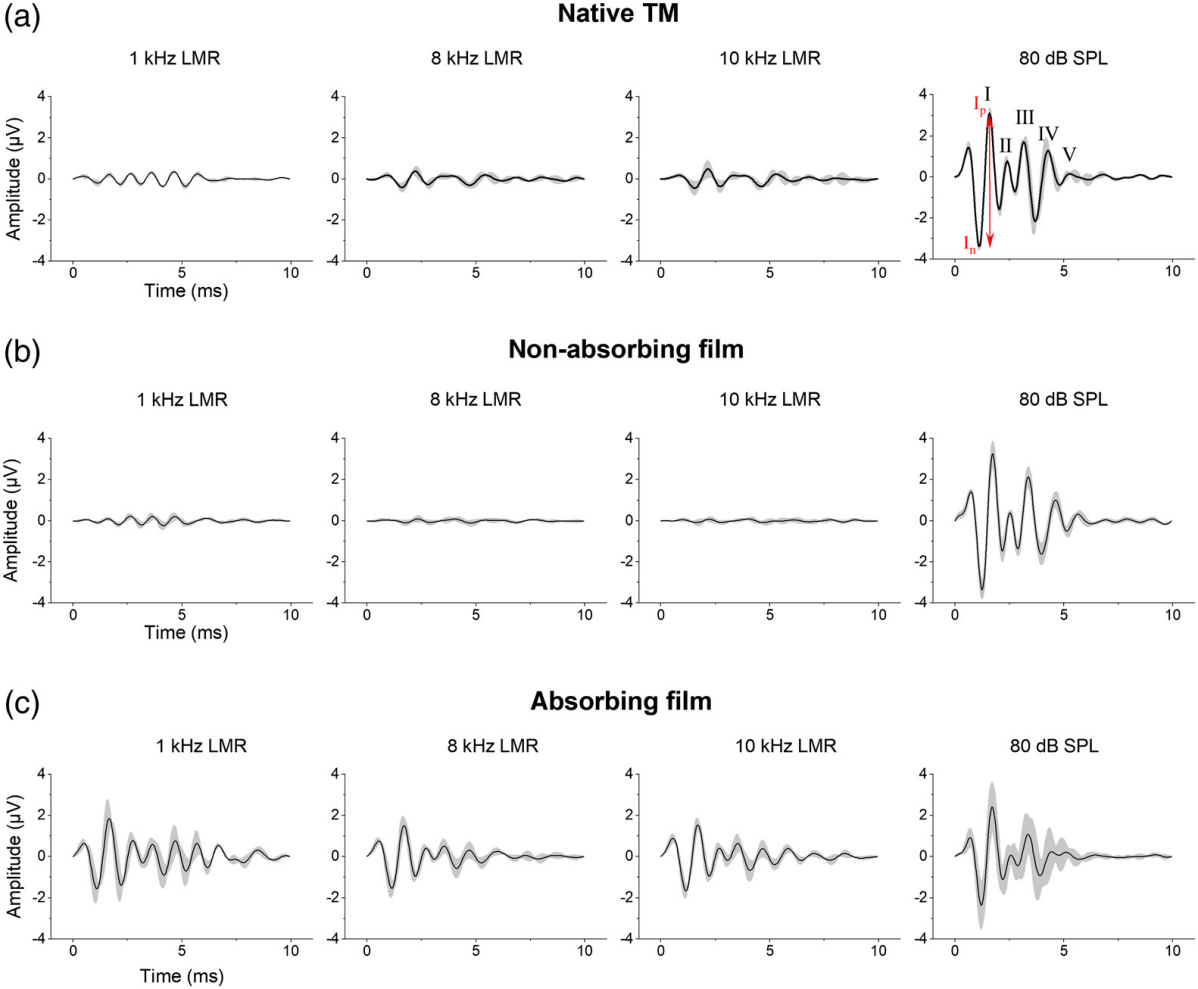
Resulting averaged oABR waves after stimulation with 79 mW average laser power (a) without a film, (b) with a nonabsorbing film, or (c) an absorbing film with 1, 8, and 10 kHz LMR in comparison to the aABR waves at 80 dB SPL acoustical stimulation (outer column), respectively. In the outer column in (a), the Jewett wave complex of wave I to V is illustrated exemplarily for all ABR waves. Wave I amplitude was analyzed from the first negative (In) to the first positive (Ip) peak [red arrow, (a) outer column]. All waves are illustrated as averaged between all replicas in the respective groups with the standard deviation. The number of replicas was (a) n=3, (b) n=5, and (c) n=6 animals.

To examine whether the application of absorbing films influences (i) the generation of optically induced ABR waves itself and (ii) the form of the resulting waves, we compared optically induced ABR waves after stimulation with 79 mW average laser powers at 1, 8, and 10 kHz LMR with acoustically induced ABR waves after stimulation with 80 dB SPL (Fig. 4). In this study, three different groups were investigated. The first group (n=3) served as a control and was stimulated on the bare TM. The second group (n=5) with a nonabsorbing film demonstrated the impact of the film on the stimulation. The third group (n=6) using an absorbing film displayed the effect of an extra absorbing layer.

We were able to induce oABR waves in all groups. The oABR waves of the control group without the use of any film [Fig. 4(a)] demonstrated clearly identifiable signals with five positive peaks, resembling the Jewett wave complex^25^^–^^27^ when stimulated with 8 and 10 kHz LMR; however, with considerably lower amplitudes when compared with the activation induced through acoustic stimulation. At 1 kHz LMR, a periodical oscillation with a frequency of 1 kHz could be detected demonstrating five waves as well, however, resembling the shape of the stimulus. The signal level was further reduced in animals that had a nonabsorbing film [Fig. 4(b)] attached to the TM, meaning that just signals at 1 kHz were identifiable. As presumed, the oABR wave complex significantly increased after the application of an absorbing film on the TM of those mice [Fig. 4(c)]. In this group of mice, the oABR wave complex was clearly identifiable after stimulation with all LMRs and the signal form and amplitude resembled that of the aABR after click stimulation with 80 dB SPL.

**Fig. 5:**
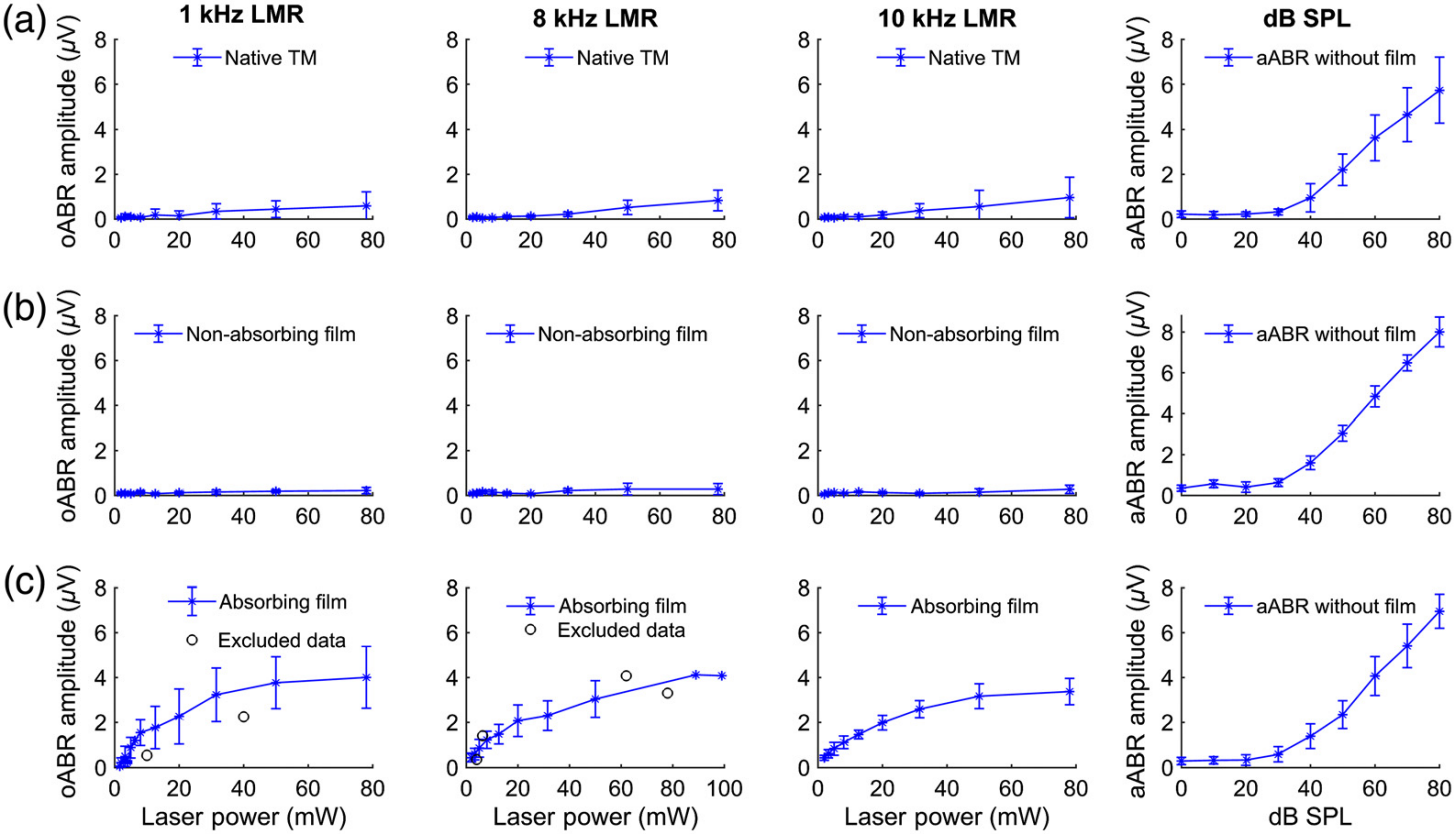
Averaged oABR amplitudes (a) without a film, and with (b) a nonabsorbing film, (c) or an absorbing film with 1, 8, and 10 kHz LMR in comparison to the averaged click stimulated aABR amplitudes (right column) of the respective group.

To analyze these differences in the signal formation, we analyzed wave I of all oABRs and compared its amplitude with the generated aABR signals in all groups. The click aABR amplitudes (Fig. 5, outer column, 0 to 80 dB SPL) followed the shape of a sigmoidal function (Fig. S1 in the Supplementary Material). The oABR stimulation (mostly 2 to 79 mW) demonstrated increasing amplitudes for rising laser power (Fig. 5). Amplitudes measured for stimulation of native TM increased linearly within the applied laser power values. Applying a nonabsorbing film reduced the amplitudes at 79 mW to ∼33% of the amplitudes recorded from the laser stimulation on a native TM. The optical stimulation of TMs with an absorbing film led to a logarithmic growth of the amplitude being 6.8 times higher at 79 mW and 1 kHz LMR, 4 times higher at 8 kHz, and 3.5 times higher at 10 kHz than the amplitudes after stimulation onto the native TM (Fig. 5).

**Fig. 6:**
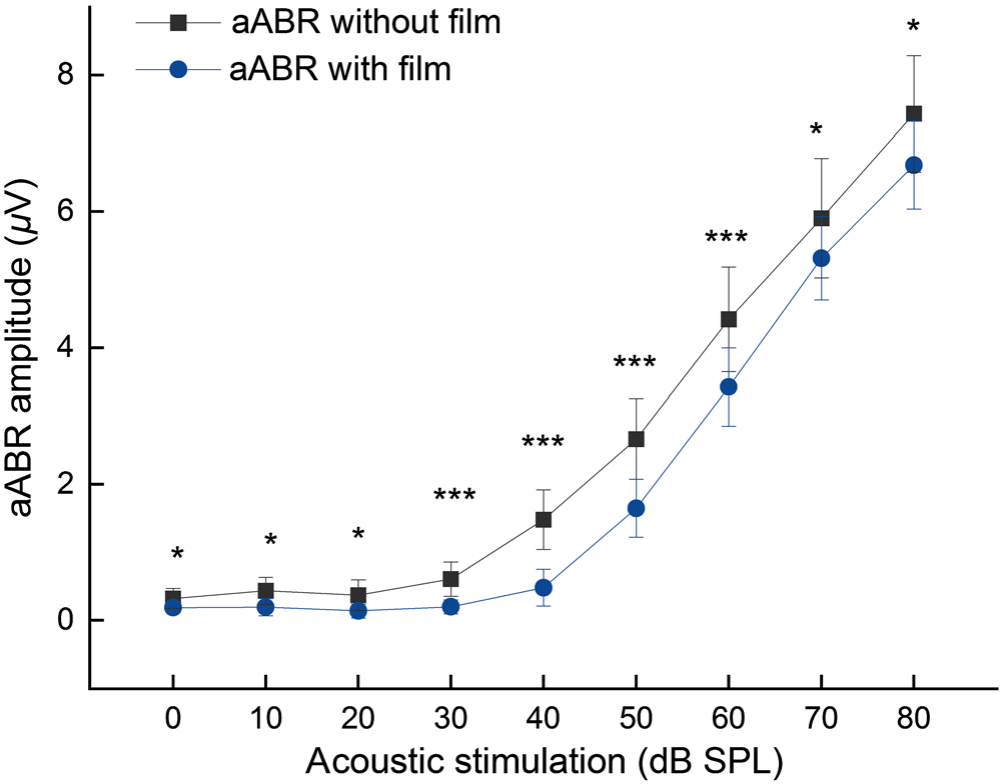
Averaged aABR amplitudes before and the lower averaged aABR amplitudes after (n=11) film application. The error bars represent the standard deviation. * indicates p<0.05; *** indicates p<0.001.

**Fig. 7:**
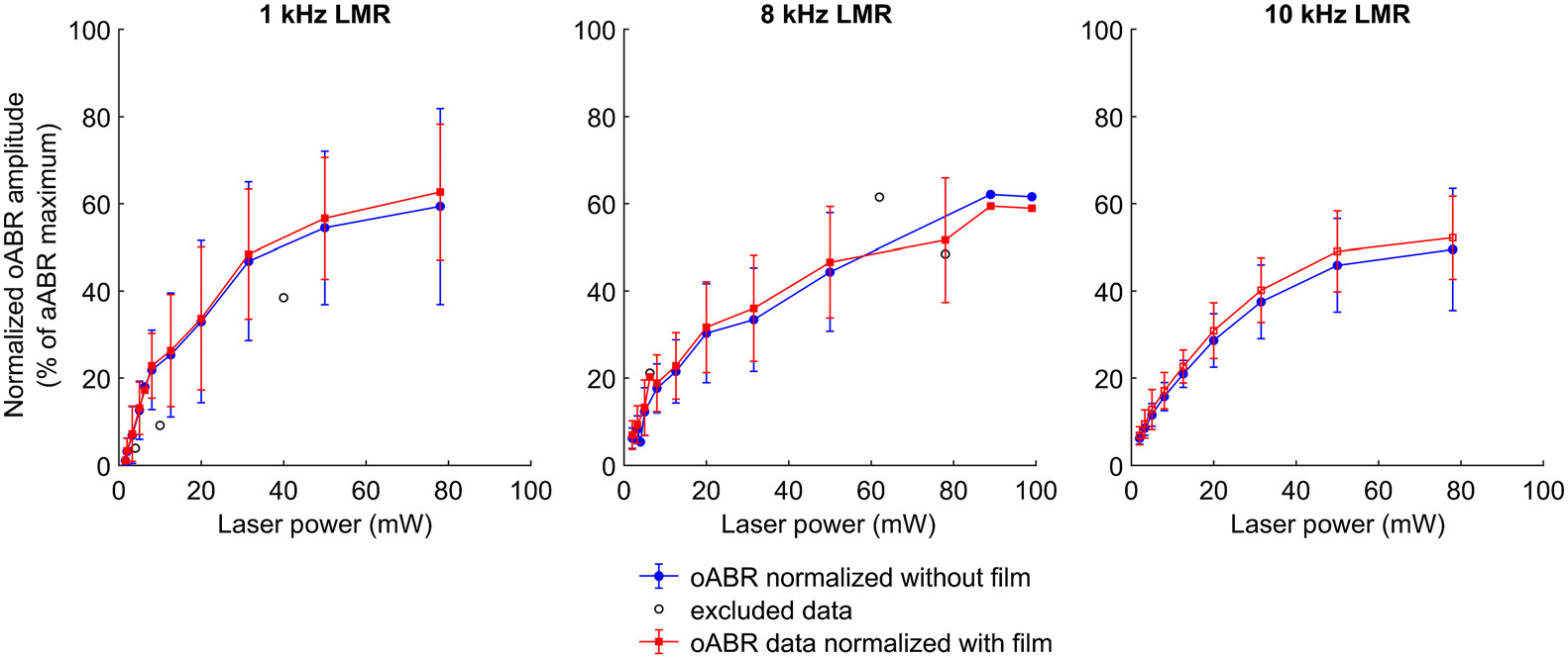
Averaged oABR amplitudes at 1, 8, and 10 kHz LMR normalized to the aABR amplitude at 80 dB SPL measured without film (blue lines) and with film (red lines) demonstrating a growth behavior with a sharp slope followed by saturation. The error bars represent the standard deviation (n=6) of the mean.

Since the aABR amplitudes without the film represented the individual hearing ability (Fig. 6) and the aABR amplitude recorded with a film attached to the TM represents the hearing ability affected by the weight of the film, both curves in Fig. 6 demonstrated the classical growth behavior known for click ABR waves. At all stimulation levels, the amplitude values were significantly lower in the group recorded with a film added to the TM. To analyze the impact of the animal’s individual hearing ability, the measured oABR amplitudes from each animal were normalized to its aABR amplitude at 80 dB SPL. To analyze this effect further and to avoid incorrect analysis of oABR recordings, we normalized the oABR wave I amplitudes to both different aABR levels (with and without film) and compared the resulting growth curves (Fig. 7). Since without absorbing film application or using a nonabsorbing film, only low intensity oABR waves could be induced, we focused in the following analysis on oABR waves after application of an absorbing film.

Following the results of the averaged wave I aABR amplitudes of all groups (Fig. 7), the wave I amplitudes normalized to the wave I aABR amplitude recorded for stimulation with 80 dB SPL after patch application demonstrated slightly higher amplitudes and smaller error bars in comparison to the oABR amplitude normalized to the aABR amplitude recorded for stimulation with 80 dB SPL before patch application. However, the difference between the amplitude values normalized with the two normalization methods in the amplitude values was <5%. Based on our data, we demonstrated that single frequency stimulation with laser power of 79 mW reached 60% of the wave I aABR amplitude level at 80 dB SPL for 1 kHz LMR, and ∼50% for 8 and 10 kHz LMR in this animal model with our current stimulation paradigm (Fig. 7).

### 3.3 Comparison of oABR and aABR Amplitudes

**Fig. 8:**
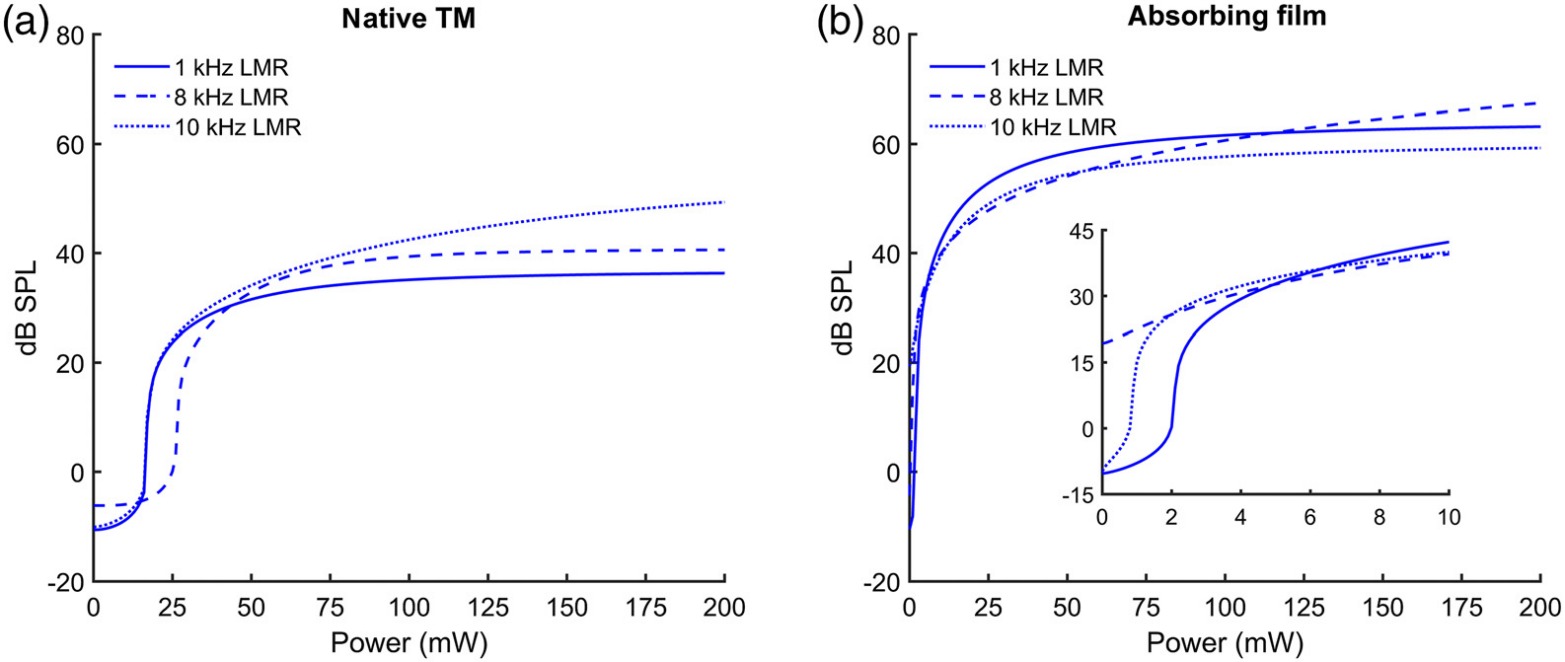
IO function [Eq. (3), Supplementary Material] calculated for the averaged data measured with (a) native TM and (b) the absorbing film for 1, 8, and 10 kHz LMR, respectively, demonstrating the sigmoidal growth behavior adopted from physiological acoustic stimulation. The absorbing film IO function is zoomed in from 0 to 10 mW.

To determine the theoretical laser power and SPL values inducing the same amplitude values, the averaged oABR and aABR amplitudes of the two groups, absorbing film, and native TM were fitted and set equal (Supplementary Material). The dynamic range of the IO function of the absorbing film was close to 70 dB SPL and, therefore, 20 dB SPL higher than the dynamic range of data recorded from irradiated native TM (Fig. 8). The turning point of the functions calculated for the group absorbing film was between 1 and 2 mW and for native TM 17 mW (1 and 10 kHz) and 27 mW (8 kHz).

## 4 Discussion

Short, pulsed laser light irradiating a medium, the TM, in this case, induces ultrasonic vibrations arising from photon absorption causing a thermal expansion and contraction of this medium.^28^ These ultrasound-induced mechanical vibrations can be used to stimulate the hearing organ at different loci.^5^^–^^9^ Therefore, the idea to work with this stimulation method to specifically influence the auditory activation suggests the design of a new generation of hearing prosthesis. A stimulation strategy based on a single laser wavelength for frequency-specific stimulation has already been demonstrated by Stahn et al.^8^ The modulation of the stimulation intensity was the next step of our research and the focus for this report.

Since we planned to explore this in the same animal model in which we performed the first biocompatibility studies^10^ to build upon those results, as a proof-of-principle study, we had first to assess if the induction of optoacoustic ABR waves is possible in mice as well since these mammals have a very transparent TM.^21^ To increase the absorption of photons additionally, we explored the effects of the application of our self-designed light-absorbing PDMS film onto the TM and investigated whether such materials could optimize the optical stimulation effects.

The detection of ABR waves is a well-established method to monitor the neural response to the stimulation of the auditory system and includes the impact of the cochlear amplifier. oABR waveform and growth behavior analysis helps to characterize the light-induced activation of the auditory pathway. Growth behavior as a function of irradiation power gives therefore an insight into the efficiency of the stimulation method represented herein by the laser irradiation. Wenzel et al. demonstrated optoacoustic stimulation-induced oABR waves in guinea pigs and used wave V growth behavior to compare the efficiency of optic stimulation in comparison to acoustic stimulation. Thereby, wave V increased with increasing laser intensities and reached a saturation plateau around 15  μJ/pulse for a 10-Hz repetition rate. The shape of wave V growth function was similar for the optoacoustic and acoustic stimulation. In our presented study, we performed optoacoustic stimulation in normal-hearing mice. Although oABRs recorded in mice were described earlier associated with optogenetic stimulation^29^ or infrared neuronal stimulation,^30^ we demonstrated, to the best of our knowledge for the first time in the literature, optoacoustically induced oABRs in mice.

Using pulsed green laser light applied on the TM, the induced ABR waves resembled, in their shape and amount of positive and negative peaks, the aABR signals. We were able to generate oABR signals with different levels on three tested conditions: applying laser light on (i) the native TM, (ii) covered TM with a nonabsorbing film, and (iii) using an absorbing film. These findings were reproducible applying different LMRs (8 and 10 kHz) (Fig. 4). At 1 kHz the auditory function in these mice was very limited and no signals should be recordable. The shape of the recorded signal at 1 kHz LMR can be compared to cochlear microphonics (CM) mimicking the stimulus [Figs. 4(a) and 4(b)].^31^ However, CMs result from the activation of the cochlea and therefore cause overlaying ABR signals as well. The excitation of the hearing system leading to a broad activation of the cochlea could also be induced by harmonics originating from irradiation of the structure behind the TM of mice, e.g., the otic capsule. This might be induced especially during stimulation without an absorber as the native murine TM or the nonabsorbing film is nearly transparent. Interestingly, oABR signals at 1-kHz LMR and an absorbing film having the classic Jewett wave shape could be detected even though, physiologically mice prove low hearing ability at this frequency. This effect might be induced through the modulation or damping of the vibration characteristics of the TM by the attached absorbing film. Further planned experiments will give more insight in this regard, specifically considering the frequency-specific activation by optoacoustic stimulation in the mouse model. In addition, the studies exploring the vibratory characteristics of the film-membrane complex, e.g., with laser Doppler vibrometry would give more insight into the sound conduction characteristics of this new stimulation method.

We used the sigmoidal growth function of wave I, the most prominent wave in murine ABR signals^1^^,^^4^^,^^32^^,^^33^ as a marker determining the intensity of a stimulus, to analyze the efficiency of optoacoustic stimulation. The optoacoustic stimulation depends on the absorption of light energy and is, therefore, a function of the absorption coefficient. The film demonstrated good light-absorbing properties (Fig. 3) and therefore increased the efficiency of the optoacoustic stimulation. This was demonstrated by the wave I amplitudes, which were enhanced by factors of 6.8, 4, and 3.5 in comparison to the irradiation of native TM. The different maximum amplitudes of the LMRs occur through the characteristic of our stimulation paradigm. By varying the LMR and keeping the LPR constant at 50 kHz fewer pulses are included in one sine period of the LMR at higher frequencies. In addition, a higher absorption coefficient does not automatically lead to higher oABR amplitudes. In their study, Kallweit et al.^34^ described an absorption coefficient optimum and a negative correlation between optoacoustic signal amplitude and absorption coefficient beyond this optimum *in vitro*.^34^ Therefore, we hypothesized and demonstrated herein that an extra absorbing layer is a solution to increase the induced activation of the auditory system.

To analyze the effectiveness of our stimulation method further, oABR amplitude values were normalized to aABR values at 80 dB SPL click stimuli. We then analyzed the impact of the film application on the wave I amplitude in aABR and found significantly reduced amplitude values when using a film attached to the TM. This finding is most likely due to the additional mass and damping induced through it on the TM, reducing therefore the sound transduction and damping the vibrations.

As a comparison, we used both aABRs, recorded with and without absorbing film, to normalize the resulting oABR wave I amplitudes (Fig. 7) and the difference was only 5%. Therefore, the impact of the conformation of the film on the interpretation of the results was for this set of experiments negligible.

The optoacoustic stimulation at 79 mW average laser power and 1-kHz LMR led to amplitude values of ∼60% of the level that could be reached with 80 dB SPL acoustic click stimulation. At this point, one should consider as well that the click activates multiple frequencies inducing a stronger ABR signal due to the summation of activated potentials in comparison to the single frequencies activated with a frequency-specific stimulation strategy (S2). Nevertheless, we opted for this method of normalizing our data to be in line with research protocols reported by other groups.^29^^,^^35^^–^^37^ Using 8- and 10-kHz LMR, the resulting level was only 50% of the click stimulation while keeping in mind that the number of pulses in one period is lower the higher the LMR. Therefore, the comparison of the oABR data with an adapted acoustic stimulus is not perfect at this point and will be optimized in future experiments.

For biocompatibility reasons with these particular laser parameters,^10^ the irradiation level was limited to 79 mW, so higher laser values and the resulting level of reached amplitude levels were simulated by fitting the resulting growth curves. Thereby, we could detect a dynamic range of 50 dB SPL when irradiating the native TM and 70 dB SPL when using an absorbing film. The fitted data also demonstrated that using our actual stimulation method, a saturation level would be reached at 80 mW, inducing amplitudes around 60 dB SPL acoustic click stimulation depending on the used LMR. Further work to optimize the optoacoustic stimulation is therefore planned. Experiments regarding the frequency-specific activation of the hearing function in mice are also intended for the future.

The self-adhesive property of the light-absorbing film and its ability to amplify the optoacoustically induced vibrations make it a promising candidate for enhancing stimulation on different application loci, e.g., TM, middle ear. The light transmission measurements demonstrated that our self-designed film would be applicable for other wavelengths as well. The tight contact between the film and the TM allows the transfer of vibrations directly to the vibrating structure, e.g., TM. Although the weight of the film on the membrane dampens the vibrations in comparison to a native TM (Fig. 6), this influence was minor regarding the effectiveness of the optoacoustic stimulation. The double-layer silicone-based design was developed to provide a structure with both good adhesion and stability. The SSA surface allows for reliable adhesion to the TM without damaging the tissue.^18^ On the other hand, the Sylgard portion provides support for the soft SSA film and creates stability during handling and application. The transmission measurements indicate how much of the applied laser energy is transmitted to the TM, which needs to be considered in the biocompatibility studies.^10^ The low transmission of the absorbing film confers increased safety while using it during optoacoustic stimulation since a very low amount of the irradiation energy would be transmitted to deeper layers, e.g., in this experimental design the middle and/or inner ear. In addition, the absorption spectrum of the two films was measured and indicates, for the absorbing film, good absorption properties for all wavelengths from 200 to 1300 nm. In contrast, the nonabsorbing film demonstrated no significant absorption and reflection from which it can be concluded that the optoacoustic effect occurs indeed in the absorbing layer of the film.

Fischer et al. demonstrated that PDMS films securely adhere on rough surfaces and even on human skin.^17^^,^^38^^,^^39^ Since our absorbing films could be manufactured in every conceivable design and structure, also other application loci, e.g., the ossicles or the otic capsule (the cochlea wall) within the middle ear, would be imaginable to serve as stimulation loci and adapt therefore to the different pathologies of the hard of hearing patients (e.g., malformed middle ears or having changed anatomy due to infections, cholesteatomas, and/or surgeries). In these cases, the optoacoustic stimulation could be applied on the residual ossicles or the inner ear wall, the otic capsule, directly. Since the optical energy in the form of laser light can be applied very focused on the targeted structure, the optoacoustic stimulation offers a very precise activation method that can be applied according to the individual needs without the very impairing occlusion effect as in conventional hearing devices or a tight contact to the vibratory structure as in bone conduction hearing devices or middle ear implants. As in a future clinical application, gains of up to 75 dB or above for people with profound hearing loss would have to be accomplished by an optoacoustic hearing aid, further strategies to optimize the stimulation strategy are needed, within biocompatibility margins.

Further design optimizations of the absorbing film are possible. For example, a completely pigmented patch could achieve even better results. In addition, the absorbing films have thermal insulating properties, which make the stimulation less susceptible to heat transmission to the vibrating tissue, and therefore increasing the biocompatibility of the stimulation method. Determination of these properties, as well as long-term application of the absorbing films on the TM, is the subject of our further investigations.

## 5 Conclusion

The optoacoustic stimulation induces oABR waves in mice that are comparable in form and amplitude to acoustically induced waves. The amplitudes obtained were considerably improved by the application of light-absorbing PDMS films on the TM. Therefore, this method is a promising approach for the realization of optoacoustic auditory prostheses.
