# Supplementary material for: Optoacoustically induced auditory brainstem responses in the mouse model enhanced through an absorbing film
Source: J Biomed Opt. 2021 Sep 3;26(9):098001. doi: 10.1117/1.JBO.26.9.098001 (PMC8414560; doi:10.1117/1.JBO.26.9.098001)
Supplement: Supplementary file 1 [file JBO_026_098001_SD001.pdf]

**Supplementary Material**

Data fitting

To analyze the o- and aABR signals at different energy levels of the stimulus, the measured values were fitted with a sigmoidal function (Fig. 2). ABR amplitudes grow at first exponentially, pass the turning point ($x_0$) of the slope, and converges at least to a maximum value ($A_2$). This behavior is based on the neural rate coding of a stimulus[28] and the sound amplification of the outer hair cell (OHC) motility.[29]

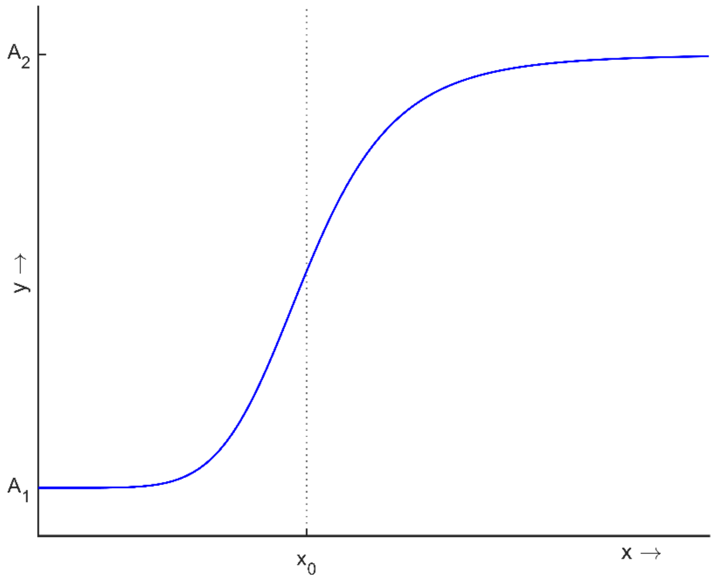


**S1** Sigmoidal fit function.

The slope of the fit function (Fig. 2) can be described with

$$y = A_2 + \frac{A_1 - A_2}{1 + \left(\frac{x}{x_0}\right)^p}, \qquad (1)$$

where $x_0$ is the turning point of the sigmoidal function, p is the growth rate, $A_1$ is the minimum value of the curve, and $A_2$ the maximum value. The dynamic range describes the y-values between $A_1$ and $A_2$ and is further used as a parameter to analyze the stimulation quality. The fits were calculated with MatLab R2019b (The MathWorks Inc., Natick, Massachusetts, USA) using our custom Equation (1) as the fit type of the program. The advantage of fitting our o-

and aABR data is the calculation of an input-output (IO) function ($y_{IO}$) [Eq. (3)] by setting the y-values of the o- and aABR fits equal [Eq. (2)]. This correlates the required optical energy to the respective click stimulated acoustic level:

$$y_{oABR} = y_{aABR}$$

$$A_{2,o} + \frac{A_{1,o} - A_{2,o}}{1 + \left(\frac{x_o}{x_{0,o}}\right)^{p_o}} = A_{2,a} + \frac{A_{1,a} - A_{2,a}}{1 + \left(\frac{x_a}{x_{0,a}}\right)^{p_a}}. \qquad (2)$$

Solving Equation (2) for $x_a$ as the new y-value and $x_o$ as x-value revealed:

$$y_{IO} = x_{0,a}\left[\sqrt[p_a]{-\frac{-A_{1,a}\left(\frac{x}{x_{0,o}}\right)^{p_o} - A_{1,a} + A_{1,o} + A_{2,o}\left(\frac{x}{x_{0,o}}\right)^{p_o}}{-A_{1,o} - A_{2,a}\left(\frac{x}{x_{0,o}}\right)^{p_o} - A_{2,a} + A_{2,o}\left(\frac{x}{x_{0,o}}\right)^{p_o}}}\right]. \qquad (3)$$

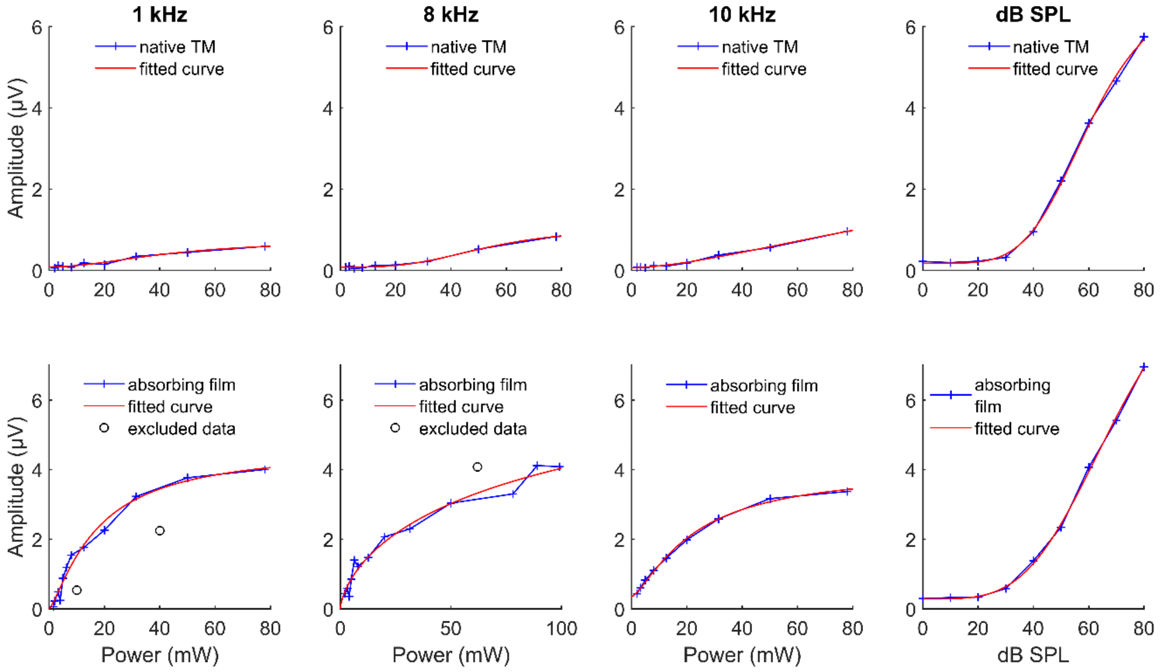


+++

**S2** Fitted curves of the averaged oABR (1, 8, and 10 kHz LMR) and click stimulated aABR (dB SPL) amplitudes of the groups, native TM (top), and absorbing film (bottom) using Equation (1).

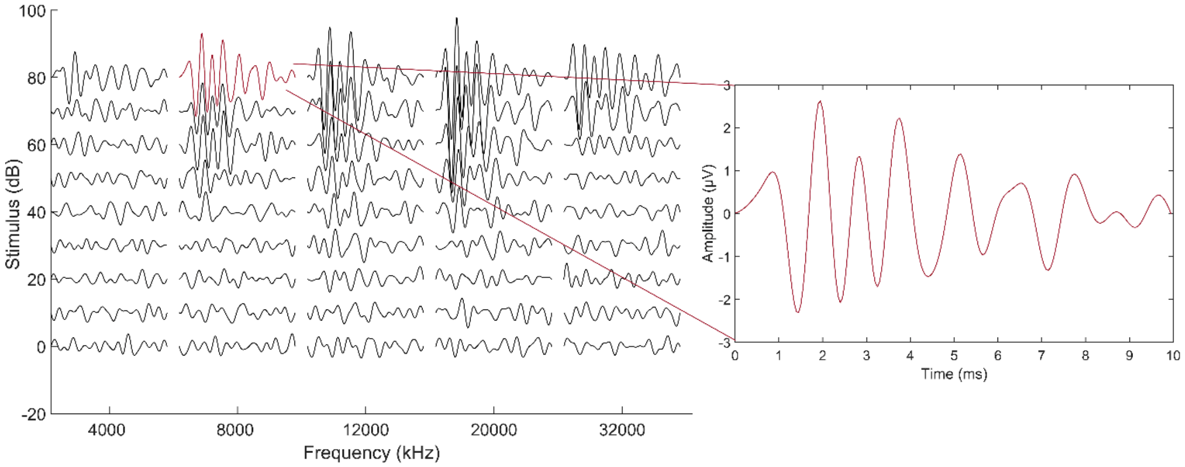


**S3** Frequency-specific ABR waves for 4, 8, 12, 20, 32, and 48 kHz acoustic stimulation from 0 – 80 dB SPL (n = 1) with a detailed plot of a curve at 8 kHz and 80 dB SPL.
